# High Correlated Paternity Leads to Negative Effects on Progeny Performance in Two Mediterranean Shrub Species

**DOI:** 10.1371/journal.pone.0166023

**Published:** 2016-11-11

**Authors:** Sofia Nora, Abelardo Aparicio, Rafael G. Albaladejo

**Affiliations:** Departmento de Biología Vegetal y Ecología, Universidad de Sevilla, Seville, Spain; National Institute for Plant Genome Research, INDIA

## Abstract

Anthropogenic habitat deterioration can promote changes in plant mating systems that subsequently may affect progeny performance, thereby conditioning plant recruitment for the next generation. However, very few studies yet tested mating system parameters other than outcrossing rates; and the direct effects of the genetic diversity of the pollen received by maternal plants (i.e. correlated paternity) has often been overlooked. In this study, we investigated the relation between correlated paternity and progeny performance in two common Mediterranean shrubs, *Myrtus communis* and *Pistacia lentiscus*. To do so, we collected open-pollinated progeny from selected maternal plants, calculated mating system parameters using microsatellite genotyping and conducted sowing experiments under greenhouse and field conditions. Our results showed that some progeny fitness components were negatively affected by the high correlated paternity of maternal plants. In *Myrtus communis*, high correlated paternity had a negative effect on the proportion and timing of seedling emergence in the natural field conditions and in the greenhouse sowing experiment, respectively. In *Pistacia lentiscus*, seedling emergence time under field conditions was also negatively influenced by high correlated paternity and a progeny survival analysis in the field experiment showed greater mortality of seedlings from maternal plants with high correlated paternity. Overall, we found effects of correlated paternity on the progeny performance of *Myrtus communis*, a self-compatible species. Further, we also detected effects of correlated paternity on the progeny emergence time and survival in *Pistacia lentiscus*, an obligate outcrossed species. This study represents one of the few existing empirical examples which highlight the influence that correlated paternity may exert on progeny performance in multiple stages during early seedling growth.

## Introduction

Mating patterns are fundamental for determining the amount and distribution of genetic variation within and between populations of plant species [1, 2]. Anthropogenic habitat deterioration may promote changes in mating patterns [3, 4], which can directly influence the genetic variation in progeny and, even more importantly, may be reflected in fitness costs that will compromise plant recruitment in the forthcoming generation [4, 5]. Mating system can be described by parameters such as selfing, outcrossing rates and correlated paternity. Numerous studies have found high variability in mating parameters such as outcrossing rates and correlated paternity levels among species [4], among populations within species [6, 7] and even among maternal plants (e.g. [8, 9, 10, 11, 12]). This variation is highly context-dependent and reflects the influence of diverse ecological factors such as landscape heterogeneity, vegetation structure and the local neighbourhood of conspecific plants [9, 10, 13]. Nevertheless, mating system parameters are mainly controlled by plant reproductive traits that permit or prevent self-fertilization (i.e. the breeding system; see [14]).

However, although numerous studies aimed at evaluating variation in mating systems within species [5, 15], only a few have ever focused on their influence on progeny performance [16, 17]. Furthermore, most current studies consider no other mating system parameter than selfing rates and overlook the direct effects of the genetic diversity of the pollen received by the maternal plants (but see [18, 19, 20]). Correlated paternity (the proportion of full-sibs within maternal progeny arrays) is an important parameter that can provide deep insights into the pollination biology of plant species [21, 22]. Pollen diversity received by the maternal plants may be advantageous for progeny fitness because it can promote pollen competition, thereby allowing post-pollination selection for those males with the fastest-growing pollen tubes [23, 24], and/or may increase female choice by post-pollination mechanisms occurring both before ovule fertilization and during seed development (as cryptic female choice) and enable them to select between progeny that differ in quality and/or compatibility [23, 24].

In this study, we analysed how correlated paternity affects progeny performance in two common shrub species, *Pistacia lentiscus* L. (Anacardiaceae) and *Myrtus communis* L. (Myrtaceae) (*Pistacia* and *Myrtus*, hereafter). Even though their life-history traits are similar in a number of ways (e.g. both are long-lived and their seeds are dispersed mainly by birds), they differ in their breeding and pollen dispersal systems. *Myrtus* has a mixed-mating system with hermaphroditic flowers pollinated by insects, while *Pistacia* is a wind-pollinated dioecious species (and thus an obligate outcrosser). The mating systems of both species have been extensively studied. For instance, Albaladejo et al. [25, 26] found a wide range for correlated paternity values in *Pistacia* linked to significant spatiotemporal variation (from 0.03 to 0.23), which, to some extent, could be attributed to inherent individual factors (such as phenological synchronisation) or the local neighbourhood of conspecific plants. Additionally, *Pistacia* is capable of extensive pollen movement and high rates of pollen flow [25]. Regarding *Myrtus* pollen dispersal, less information is known. *Myrtus* populations often show very high levels of correlated paternity (in the range 0.40–0.61; [11, S. Nora, unpublished results]). Moreover, variation in outcrossing rates in *Myrtus* populations (from 0.13 to 0.62) has been shown to significantly affect progeny performance, with higher outcrossing rates being associated with higher seedling emergence and survival under greenhouse conditions [27]. However, to date no attempts have been made to test the impact of correlated paternity variation on progeny performance in these two species.

Here, we combined mating system analysis with data on progeny performance measurements based on both greenhouse and natural field sowing experiments. Specifically, we aimed (1) to assess the influence of correlated paternity on early progeny performance (here evaluated as seedling emergence, seedling emergence time, seedling growth, biomass, lifetime and survival) in *Myrtus communis* and *Pistacia lentiscus*, and (2) to verify the consistency of the observed relationships both in a greenhouse environment and under natural (field) conditions, where they could be potentially masked by other factors such as the response to environmental heterogeneity.

## Material and Methods

### Ethics statement

Permission for conduct this study was obtained from the *Delegación Provincial de la Consejería de Medio Ambiente de la Junta de Andalucía*, Spain. This study did not involve endangered or protected species.

### Study species and system

*Myrtus communis* and *Pistacia lentiscus* are widespread and abundant shrubs in sclerophyllous Mediterranean woodlands. In the study area, *Myrtus* grows up to 4 m in height and blooms massively in early summer (from mid-June to early July) and its white flowers are hermaphroditic and pollinated mostly by hymenopterans and dipterans [28]. *Myrtus* fruit are berries (mean ± SD = 5.2 ± 2.7 seeds; [27]) that turn dark blue when mature. Fruits mature from mid-October to late November and seeds are dispersed by passerine birds, mostly Sylviidae and Turdidae [29]. *Pistacia* is a dioecious shrub up to 4 m in height. It is wind-pollinated and blooms massively between mid-March and late April [30]. Its fruits are small black one-seeded drupes and mature in September–December and are consumed by a similar guild of dispersers as those of *Myrtus* [31].

**Fig 1:**
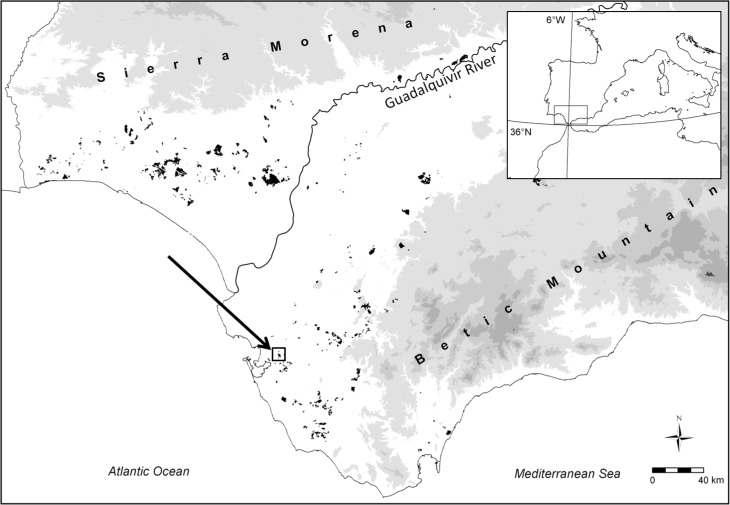
Map of the Guadalquivir River Valley (southwestern Spain), the location of the stone pine forest selected as a study area (Dehesa de las Yeguas). The unshaded area represents lowland areas (< 200 m above sea level), the remaining woodland patches are shaded in black and higher elevations and legally protected areas are in light and dark grey, respectively. The arrow indicates the location of the study area.

This study was conducted in the Guadalquivir river valley (southwestern Spain), a large (21,000 km^2^), fertile and intensively cultivated Mediterranean lowland. This region has a long history of human intervention and management [32] and remnant woodland patches cover less than 1% of their potential area [33]. The study area, Dehesa de las Yeguas (36°33'15''N, 6°08'08''W), is ca. 100 hectares of semi-natural stone pine (*Pinus pinea* L.) forest (Fig 1). In the study area, shrub cover represents 63.82% of the total study area and is mainly composed of small (<1 m) dry-fruited shrubs (Cistaceae, Lamiaceae and Leguminosae) and tall (>1 m) fleshly fruited shrubs, amongst which *Pistacia* and *Myrtus* are the dominant species. The climate is typically Mediterranean, with a mean annual precipitation of ca. 650 mm and an intense summer drought (approximately 10 mm rainfall in July and August), and a mean monthly temperature of 19°C (ranging from 9°C in February to 25°C in August) (data from *subsistema CLIMA* available at http://www.juntadeandalucia.es/medioambiente).

In November 2010, we collected naturally produced mature fruits from throughout the canopy of 18 *Myrtus* and 18 *Pistacia* maternal plants. Study plants were selected to cover the range of natural heterogeneity in the local environment (mainly plant density, from clumped to isolated plants). Therefore, local variation was chosen *a priori* to try to capture the highest variability in mating system parameters at individual plant level [9, 10, 13, 24].

### Microsatellite genotyping and individual mating system analyses

For genetic analysis we randomly selected 16 seeds per maternal plant. *Pistacia* embryos were easily excised from the maternal endocarp and endosperm, so they did not require germination. In *Myrtus* only one seed per fruit was used to avoid the effects of correlated pollination in a single pollination event. Seeds from each maternal plant were germinated in separate Petri dishes containing filter paper saturated with distilled water. Previous studies indicate that germination rates in *Myrtus* are very high [11] and in this study almost all seeds germinated within a few days (mean germination rates ± SD = 95.0% ± 6.9%). For DNA isolation, *Myrtus* seedlings were harvest once both cotyledons were fully developed.

We extracted DNA from *Myrtus* seedlings and *Pistacia* embryos with the Invisorb DNA Plant HTS 96 Kit (Invitek, Germany) following the manufacturer’s protocol. We amplified seven and eight polymorphic nuclear microsatellites in *Pistacia* and *Myrtus*, respectively. Further details regarding the amplification reactions and PCR cycle conditions can be found in Albaladejo et al. [34, 35] and Nora et al. [36]. We analysed fluorescently labelled (with 6-FAM, NED, VIC and PET dyes) PCR products with an ABI 3730 DNA Analyzer (Applied Biosystems, Foster City, US) at the *Unidad de Genómica* (UCM, Madrid, Spain). Fragment sizes were automatically scored with GeneMapper 3.7 (Applied Biosystems, Foster City, US) and corrected manually when necessary. We only retained multilocus genotypes that successfully scored with at least four loci. Maternal genotypes had already been analysed for a companion study [36]. We checked for genotyping errors by identifying mother-offspring mismatches and obtained an overall mismatching estimate of 1.90% for *Myrtus* and 2.14% for *Pistacia*.

Independence among pairs of loci of maternal genotypes was checked through linkage disequilibrium (LD) tests using the Genepop web server (http://genepop.curtin.edu.au) and tested for null alleles with Micro-Checker [37]. We calculated maternal genetic diversity levels via the homozygosity by loci (*HL*) index, a calculation that improves raw heterozygosity estimates by weighting the contribution of each locus to the individual homozygosity value in terms of their allelic variability [38]. We calculated *HL* with the R script Genhet [39]. We estimated the multilocus (*t*_*m*_) and single locus (*t*_*s*_) outcrossing rates and the correlated paternity (*r*_*p*_) for maternal plants with the software Mltr 3.4 [40]. Pollen and ovules were constrained to have the same gene frequencies and standard errors, and confidence intervals were assessed by bootstrap (1000 replicates) with individuals within families as the resampling unit. For *Pistacia* (as this species is dioecious), we used only the parameters *t*_*m*_ and *t*_*s*_ to estimate the biparental inbreeding (*t*_*m*_*-t*_*s*_, i.e. mating between genetically related individuals).

### Progeny performance under greenhouse conditions

In April 2011, we individually sowed a total of 720 seeds (20 seeds from each maternal plant of both species) in trays of 60 pots (5 x 5 cm and 17 cm in depth). We filled each pot with horticultural blend (5:1 peat and perlite) and sowed the seeds at a depth of c. 0.5 cm. We watered the trays twice a week and randomly changed their position every 2–3 weeks to ensure homogeneous conditions inside the greenhouse throughout the 424 days of the experiment. We regularly monitored ‘seedling emergence’ (every 2–3 days for two months after sowing) and ‘seedling mortality’ until the end of the experiment. A seedling was regarded to have emerged when it extended cotyledons. For each emerged seedling we also recorded the following data: (1) ‘seedling emergence time’ as the number of days between sowing and seedling emergence (monitored every 2–3 days for two months after sowing), (2) ‘seedling growth’, measured as seedling height at four dates distributed regularly throughout the experiment (days 36, 113, 190 and 267) and including a final measurement at the end (day 424), and (3) ‘seedling biomass’ measured as the dry weight at the end of the experiment. To measure the biomass, after harvesting we dried the surviving plants at 60° C for 72 h and, in order to obtain accurate measurements of the biomass allocation, we separately measured the shoot and root dry weight of each plant.

### Progeny performance under field conditions

We performed a sowing experiment using 30 seeds from each maternal plant (totalling 1080 seeds) to assess seedling performance under natural conditions in Dehesa de las Yeguas (Spain). To avoid bias between seedling emergency of sowed seeds of our experiment and seedlings from natural dispersed seeds, we started this experiment in early March 2011, the end of the natural dispersion period (cf. González-Varo et al. [41]). We sowed seeds in three distinct microhabitats: (1) under conspecific shrubs (*Myrtus* seeds under *Myrtus* shrubs and *Pistacia* seeds under *Pistacia* shrubs), (2) under heterospecific shrubs (*Myrtus* seeds under *Pistacia* shrubs and *Pistacia* seeds under *Myrtus* shrubs), and (3) in open ground under tree cover. We focused our experiment on these microhabitats because they are traditionally considered suitable places for birds to drop seeds and are consequently favourable places for seedling recruitment (e.g. [41, 42, 43, 44]). We established 10 sowing plots per microhabitat (30 plots in total). In each plot, we removed any naturally dispersed seedlings and sowed uniformly one seed from each of the selected maternal plants (i.e. 18 seeds of each species) at a depth of 0.5–1 cm. To match natural conditions as close as possible, we then added a thin layer of litter on top of the seed after sowing. Each plot was protected by a wire mesh cage (with an upper surface grid area of 15 × 10 cm and 10 cm in height) to prevent predation by rodents.

We conducted this experiment and monitored each emerged seedling until September 2011 (i.e. after the first summer). We monitored ‘seedling emergence’ and ‘seedling mortality’ weekly for three months and fortnightly thereafter until the end of the experiment. Like in the greenhouse, in this field experiment, seedlings were regarded to have emerged when they fully extended cotyledons. For each seedling we also recorded the following variables: (1) ‘seedling emergence time’, as the number of days between sowing and seedling emergence and (2) ‘seedling lifetime’, as the total number of days a seedling lived from emergence until death or until the end of the experiment (for those seedlings that survived). To control soil moisture, we used a time domain reflectometer (TDR, Campbell Scientific Inc., Logan, UT, USA) with 12-cm depth rods to measure soil volumetric water content (VWC; %). This measure is fundamental because the water deficit, especially during the summer, is a critical factor for seedling survival in many Mediterranean plant species (e.g. [42, 44]). Measurements were taken at an adjacent point (~20 cm) to each sowing plot every two weeks in the first three months and monthly thereafter.

### Statistical analyses

For each species, we compared seedling performance between experiments (greenhouse vs. field) using the non-parametric Wilcoxon paired test. Correlations between seedling height and seedling total dry biomass, as well as between shoot and root dry biomass were made using Spearman’s correlations. We reported data as means ± SE.

We evaluated the relationships between the correlated paternity (*r*_*p*_) and progeny performance measurements using generalized linear models (GLMs) in R 3.0.2 (R Developmental Core Team 2016). We conducted all analyses at maternal plant level and used a gamma distribution with a log link function for modelling the response variables. To check whether *r*_*p*_ was correlated with other mating system variables we performed Spearman’s correlation tests and observed no significant correlations between correlated paternity and any other genetic parameter (*HL*, *t*_*m*_*-t*_*s*_ or *t*_*m*_) neither in *Myrtus* nor in *Pistacia* (S1 Table). We accounted for the effects of seed mass on the response variables ‘proportion of seedling emergence’, ‘seedling growth’ and ‘seedling lifetime’, and the effects of ‘seedling emergence time’ on the variables ‘seedling growth’, ‘seedling biomass’ and ‘seedling lifetime’ by including these variables as covariates in the models. To obtain the mean seed mass for each maternal plant we randomly sampled 20 seeds per maternal plant (one seed per *Myrtus* fruit) and weighed it to the nearest 0.1 mg.

We evaluated seedling survival rates of *Pistacia* in the field sowing experiment with survival analyses [45] and we tested the effects of correlated paternity using Cox’s proportional hazard regression [46]. This analysis was performed with the software Statistica v.6 (StatSoft 2001).We considered seedlings that at the end of the study were still alive as censored data. No *Myrtus* seedlings survived the first summer in any of the microhabitats (see Results) and so this analysis could not be performed for this species.

## Results

### Genetic diversity and mating parameters

We genotyped open-pollinated progeny from 18 maternal plants of *Myrtus* (n = 266) and *Pistacia* (n = 283). Microsatellite markers were highly polymorphic in both species with a total of 57 and 40 different alleles identified across all maternal plants for *Myrtus* and *Pistacia*, respectively. Linkage disequilibrium tests showed that all pairs of loci in the two species were independent and null allele presence was not significant at any loci within any species.

Overall, *Myrtus* maternal plants had high levels of homozygosity (mean *HL* = 0.386 ± 0.035) and correlated paternity (mean *r*_*p*_ = 0.395 ± 0.045) (S2 Table). On the other hand, *Pistacia* maternal plants had relatively low levels of both homozygosity (mean *HL* = 0.271 ± 0.034) and correlated paternity (mean *r*_*p*_ = 0.060 ± 0.006). Both parameters presented a large variation among *Myrtus* maternal plants: *HL* ranged from 0.18 to 0.69 and *r*_*p*_ from 0.10 to 0.87. In *Pistacia* maternal plants, we also found high variability in *HL* levels, ranging from 0.00 to 0.49 but *r*_*p*_ values were more stable among maternal plants, ranging from 0.03 to 0.11. As expected in a dioecious species, estimated outcrossing rate (*t*_*m*_) in *Pistacia* was very close to one (S2 Table). The mean value of *t*_*m*_ in *Myrtus* maternal plants was 0.704 ± 0.044, ranging from 0.25 to 0.98 (S2 Table). *Myrtus* displayed relatively higher rates of biparental inbreeding than *Pistacia* (*t*_*m*_*-t*_*s*_ = 0.264 ± 0.027 vs. 0.065 ± 0.005).

### Progeny performance under greenhouse conditions

The mean percentage of seedling emergence was 53.33% (± 2.26) in *Myrtus* and 49.17% (± 1.73) in *Pistacia*. *Myrtus* seedlings took on average 20.04 (± 0.40) days to emerge (emergence time was 14–36 days after sowing), while *Pistacia* seedlings took 18.90 (± 0.40) days for emergence (range of 14–44 days) (S3 Table). Seedling mortality was low throughout the experiment in the greenhouse (15.03% in *Myrtus* and 16.38% in *Pistacia*). Approximately one month after the start of the experiment, *Pistacia* seedlings had a mean height of 26.9 (± 0.8) mm and *Myrtus* seedlings a mean of 16.7 (± 0.8) mm (S3 Table). At the end of the experiment, the mean heights were 391.6 (± 9.8) mm for *Pistacia* seedlings and 257.5 (± 9.3) mm for *Myrtus* seedlings. *Pistacia* seedlings had a final mean dry biomass of 2.87 (± 0.04) g and *Myrtus* seedlings 2.39 (± 0.04) g (S3 Table). In both species, seedling height at the end of the experiment was highly correlated with seedling total dry biomass (Spearman’s *r*_*Myrtus*_ = 0.87, *P* < 0.001, and *r*_*Pistacia*_ = 0.77, *P* < 0.001). Shoot and root dry biomass were also highly correlated in both species (Spearman’s *r*_*Myrtus*_ = 0.92, *P* < 0.001 and *r*_*Pistacia*_ = 0.71, *P* < 0.001).

**Fig 2:**
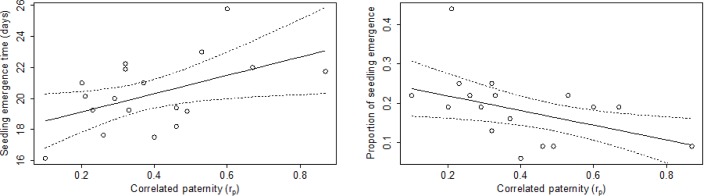
Relationships between correlated paternity and seedling performance variables for *Myrtus communis* derived from GLMs in the greenhouse (left) and field (left) experiment.

The influence of correlated paternity on seedling performance in the greenhouse environment is summarized in Table 1. Correlated paternity had no effect on *Myrtus* ‘proportion of seedling emergence’, ‘seedling growth’ or ‘seedling biomass’ but had a significant positive effect on ‘seedling emergence time’ (Fig 2). Correlated paternity had no significant effect on *Pistacia* seedling performance measurements (‘proportion of seedling emergence’, ‘seedling emergence time’, ‘seedling growth’ and ‘seedling biomass’) (Table 1).

**Table 1:** Results from generalized linear models (GLMs) examining the influence of correlated paternity on fitness measurements in seedlings of *Myrtus communis* and *Pistacia lentiscus* grown under greenhouse conditions.

Fitness measurements	Correlated paternity (*r*_*p*_)					Other covariates
	Estimate	±	SE	*t*	*P*	
*Myrtus communis*						
Emergence (proportion)	-0.480	±	1.012	-0.474	0.642	Sm*
Emergence time (days)	**-0.013**	**±**	**0.006**	**-2.190**	0.045*	Sm
Seedling height (mm)						
After 36 days	0.053	±	0.352	0.150	0.883	Sm*, Et
After 113 days	-0.039	±	0.041	-0.964	0.351	Sm, Et
After 190 days	-0.029	±	0.024	-1.208	0.247	Sm, Et
After 267 days	-0.027	±	0.023	-1.179	0.258	Sm, Et
After 424 days	-0.026	±	0.019	-1.376	0.190	Sm, Et
Dry biomass (g)						
Total	-0.009	±	0.013	-0.732	0.476	Sm, Et
Shoot	-0.021	±	0.032	-0.656	0.522	Sm, Et
Root	-0.015	±	0.018	-0.870	0.399	Sm, Et
*Pistacia lentiscus*						
Emergence (proportion)	8.423	±	8.440	0.998	0.334	Sm
Emergence time (days)	-0.079	±	0.069	-1.156	0.266	Sm
Seedling height (mm)						
After 36 days	-0.639	±	0.719	-0.889	0.389	Sm*, Et**
After 113 days	-0.008	±	0.130	-0.065	0.949	Sm, Et
After 190 days	0.005	±	0.082	0.058	0.954	Sm, Et
After 267 days	-0.013	±	0.080	-0.161	0.875	Sm, Et
After 424 days	0.007	±	0.072	0.103	0.919	Sm, Et
Dry biomass (g)						
Total	0.033	±	0.050	0.649	0.527	Sm, Et
Shoot	0.066	±	0.132	0.502	0.623	Sm, Et
Root	0.001	±	0.001	0.889	0.389	Sm, Et

Covariates: seed mass (Sm) and emergence time (Et). Significant *P*-values in bold

* < 0.05

** < 0.01.

The covariate ‘seed mass’ had a significant positive effect on ‘proportion of seedling emergence’ in *Myrtus* and in the first measurement of seedling height (‘seedling height after 36 days’) in both species (Table 1). ‘Seedling emergence time’ only had a significant effect on seedling height in *Pistacia*.

### Progeny performance under field conditions

*Pistacia* had similar seedling emergence rates in field and greenhouse environments (Wilcoxon’s test; *Z* = 0.15, *P* = 0.88). However, field conditions had a strongly significant negative effect on *Myrtus* seedling emergence (Wilcoxon’s test; *Z* = 3.59, *P* < 0.001). Both *Myrtus* and *Pistacia* seedlings took longer to emerge in the field than in the greenhouse (Wilcoxon’s test; *Z* = 3.72, *P* < 0.001 and *Z* = 3.72, *P* < 0.001, respectively) (S3 Table). *Myrtus* seedlings in the field took on average 39.19 (± 0.95) days to emerge, whereas *Pistacia* seedlings emerged slightly earlier, 34.24 (± 0.60) days.

**Fig 3:**
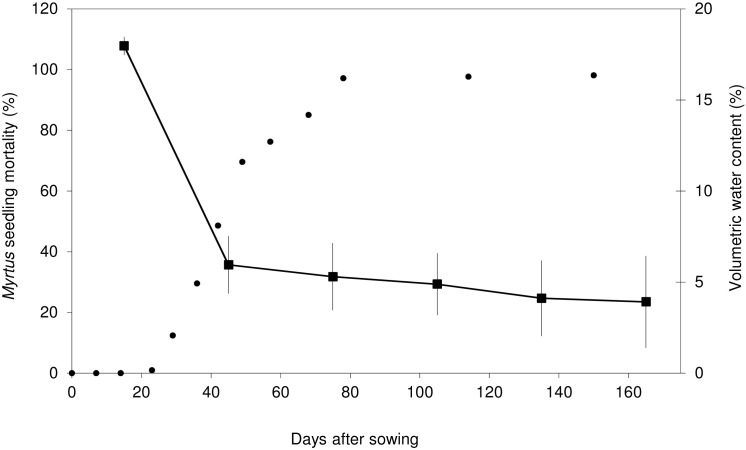
Cumulative percentage of *Myrtus communis* seedling mortality in field environment (dots) and temporal dynamics in mean soil volumetric water content (%) in the plot where sowing experiments were carried out (thin line).

We did not conduct GLMs on seedling survival due to low seedling survival in *Pistacia* (only 40 seedlings survived to the end of the experiment) and due to the lack of surviving *Myrtus* seedlings. *Myrtus* seedling mortality rate rose dramatically from 12.38% at day 29 to approximately 100% at day 78 and was clearly associated with the measured volumetric water content in the ground (see Fig 3). For this reason, the potential of seedling survival for this species was assessed indirectly by using seedlings’ lifetimes as a response variable (see Table 2).

The influence of correlated paternity on seedling performance in the field experiment is summarized in Table 2. Contrary to the effects detected in the greenhouse experiment, we found no significant trend towards the influence of correlated paternity on ‘seedling emergence time’ and ‘seedling lifetime’ in *Myrtus* under field conditions. For this species we found a significant negative relationship of correlated paternity on ‘proportion of seedling emergence’ (Fig 2). Correlated paternity had no association with *Pistacia* seedling performance measurements ‘seedling emergence’ and ‘seedling lifetime’. However, we found a significant relationship between correlated paternity and ‘seedling emergence time’ in *Pistacia* under field conditions. The covariate ‘emergence time’ had a significant effect on ‘seedling lifetime’ in *Myrtus*.

**Table 2:** Results of the generalized linear models (GLMs) examining the influence of correlated paternity on fitness measurements in seedlings of *Myrtus communis* and *Pistacia lentiscus* grown under natural field conditions.

Fitness measurements	Correlated paternity (*r*_*p*_)					Other covariates
	Estimate	±	SE	*t*	*P*	
*Myrtus communis*						
Emergence (proportion)	7.660	±	3.492	2.193	0.045*	Sm
Emergence time (days)	0.004	±	0.005	0.779	0.448	Sm
Lifetime (days)	-0.003	±	0.009	-0.335	0.743	Sm, Et*
*Pistacia lentiscus*						
Emergence (proportion)	-0.364	±	4.722	-0.077	0.940	Sm
Emergence time (days)	-0.043	±	0.020	-2.196	0.044*	Sm
Lifetime (days)	0.051	±	0.048	1.061	0.307	Sm, Et

Covariates: seed mass (Sm) and emergence time (Et). Significant *P*-values in bold

* < 0.05.

**Fig 4:**
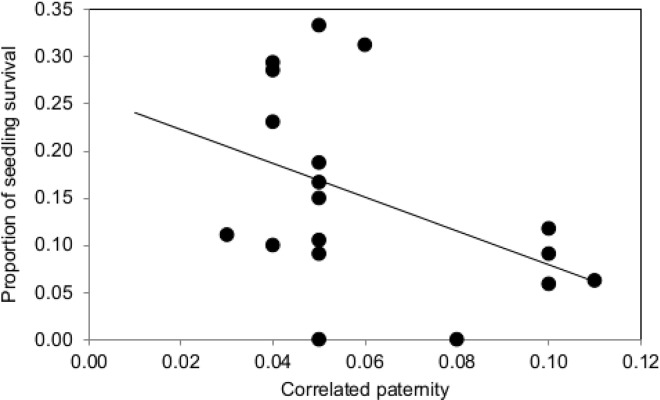
*Pistacia lentiscus* seedlings survival as a function of correlated paternity in the field environment.

Survival analysis regression test detected the influence of correlated paternity in *Pistacia* seedling survival. When running the analyses we found significant negative effect of correlated paternity (*Chi*^*2*^ = 4.09, df = 1, *P* = 0.043; Fig 4), indicating that progeny from maternal plants with high correlated paternity had lower survival rates than progeny from maternal plants with low correlated paternity.

## Discussion

Plant recruitment is an essential stage of plant population dynamics and the long-term persistence of plant populations. In order to understand plant recruitment, it is fundamental to determinate which factors are affecting the demographic processes involved in early seedling performance (e.g. seedling emergence, growth and first-year survival). Our study is one of the few to date that has investigated whether the pollen diversity naturally received by maternal plants–here assessed by the correlated paternity–has an influence on the subsequent progeny performance. To accomplish our goal, we integrated mating system analysis with data on progeny performance both in greenhouse and field-sowing experiments.

The effects of correlated paternity on progeny performance were more evident in *Myrtus*. Under greenhouse conditions, we observed that high correlated paternity was associated to a delay in seedling emergence. In Mediterranean environments early emergence is of critical importance in determining subsequent seedling growth [47], as was also observed in our study, in which emergence time influenced seedling fitness. Furthermore, the negative influence of high correlated paternity on the percentage of seedling emergence was also evident in the field environment, i.e. under more stressful conditions. These results fit the hypothesis that maternal plants that receive high pollen diversity (i.e. have low correlated paternity) generate progeny with increased fitness. As hypothesized in the literature, this relation could be due to female selection for more compatible pollen [24, 48], a process that could occur both before fertilization (e.g. by pollen germination and pollen-tube growth) or after [23, 49]. In addition, our results concur with the few other empirical studies in which the effects of correlated paternity on fitness progeny have been tested. For instance, Llorens et al. [20] reported both direct and indirect (through the increase of seed size) relationships of paternal diversity on seed germination, seedling weight and survival in *Banksia sphaerocarpa*. Also, Breed et al. [18, 19] reported negative effects of high correlated paternity on sapling growth in *Swietenia macrophylla* and *Eucalyptus socialis*.

Contrary to the results obtained under greenhouse conditions, seedling emergence time in the field was not significantly affected by correlated paternity, even though progeny from maternal plants with high correlated paternity had greater emergence times that progeny from maternal plants with low correlated paternity. We probably only observed this relationship in the greenhouse because under field conditions the influence of correlated paternity was masked by other factors that may condition this fitness variable more critically. Several studies documented the particularly importance of abiotic factors (such as water, light, temperature, microhabitats and disturbances) on seedling establishment under the harsh summer conditions of the Mediterranean climate (e.g. [41, 44]).Still, even under this type of natural conditions, we were able to detect the influence of correlated paternity in the percentage of seedling emergence in *Myrtus*.

It has been suggested that higher pollen diversity moderates the effects of inbreeding depression on progeny performance by lessening the likelihood that pollen with expressed deleterious alleles is involved in reproduction [50]. In our study, although it is difficult to completely disentangle the effects of correlated paternity from the effects of inbreeding, in some cases it was possible to detect a trend indicating that correlated paternity could have an effect on progeny fitness independently of inbreeding (whenever an effect of *r*_*p*_ independently of *t*_*m*_ or *HL* results is observed), which agrees with findings in other studies (e.g. [19]). Nevertheless, this subject still needs further research.

In *Pistacia*, no significant impact of correlated paternity on progeny performance was evident in the greenhouse conditions. *Pistacia* is an obligate outcrossed species so it is not surprising that it has low levels of correlated paternity. Thus, the inherent pollination biology of *Pistacia*, (leading to inherent low levels of correlated paternity) is diluting the potential trends that we found in *Myrtus*.

However, under field conditions we were able to detect the influence of correlated paternity on *Pistacia* seedling performance. For instance, and even though abiotic factors (especially drought) may be a major cause of the low seedling establishment in the Mediterranean [42, 44, 51], even more than genetic factors [41], our survival analyses did find some evidence of the positive influence of low correlated paternity in a very important stage of the progeny fitness: survival after the first summer. Surviving the first summer in the harsh Mediterranean conditions is critical and this period is the most likely to act as a bottleneck for plant recruitment [41, 44, 52, 53].

Under field conditions, we also observed the influence of correlated paternity in delaying *Pistacia* emergence time, a fundamental characteristic in the Mediterranean, as mentioned before. In fact, several *Pistacia* characteristics such as early seedling emergence make this species competent and able to survive efficiently in the Mediterranean climate [54]. Early emergence leads to benefits for seedling growth and fecundity, especially important in a Mediterranean environment in which species have to grow sufficiently during rainy spring to survive the summer drought [47]. The timing of emergence will in fact determine the seedling’s fate as a plant. In our study, it is likely that the early emergence of *Pistacia* seedlings (as compared to *Myrtus*) in the field coincided with more benign conditions that resembled those in the greenhouse, which may have led to the similar percentage of emergence observed in the two sowing experiments for this plant. After the first summer, only 8% of *Pistacia* seedlings survived in the field experiment, a low survival rate but higher than that of *Myrtus* (in which no seedlings survived to the end of the experiment). Summer water deficit is the main factor of stress and mortality for seedlings in Mediterranean woody plants [41, 42, 44, 51]. Nearly all dead seedlings in our field experiment were found to be desiccated (S. Nora, personal observation) and so seedling mortality is probably mostly attributable to drought. In fact, *Myrtus* seedling mortality was already 48.57% after just two months of the experiment (approximately at the end of April), by which time the measured volumetric water content in the ground had fallen dramatically. Plant species that do not form persistent soil seed banks (such as *Myrtus* or *Pistacia*) depend on a short temporal window after dispersal or on rare rainfall events to be able to recruit. Bearing in mind these conditions, the probability of natural seedling establishment in these two species–and especially in *Myrtus*–is extremely limited [41].

In this study, seedlings assessed for progeny fitness were not genotyped, which have been ideally the best approach in order to obtain direct fitness–mating system associations. However, we rather used family mating system estimates to detect associations between correlated paternity and progeny fitness. We considered this approach in order to avoid bias in the estimation of the mating system parameters, because genotyping only emerged seedlings could have biased upwardly the mating system estimates (considering that the percentage of emerged seeds in the greenhouse was ~ 50% for both species and in the field experiment the percentage was even lower). Bearing this in mind, it is fundamental to reckon that in this study the detected effects between mating system parameters and the fitness variables are indirect.

Our results on progeny fitness-correlated paternity associations found multiple non-significant effects and thus, we recognize that our results might lack significance once a multiple correction is applied. However, we considered that our study present novel results which could promote the forthcoming of new researches on the effects correlated paternity in other plant species and future meta-analysis studies.

## Conclusions

We found that the correlated paternity has an impact on different aspects of progeny performance. In this study, we report a strong influence of correlated paternity on the progeny performance of *Myrtus*, a self-compatible species. Even so, in *Pistacia* (outcrossed species) the negative consequences of high correlated paternity on progeny emergence time and survival under field conditions were also detected. We conclude that our findings, in combination with a handful of recent empirical studies, suggest that mating pattern parameters–other than outcrossing rates–influence progeny performance in multiple stages during early seedling growth.

## Supporting Information

S1 Table Correlations among maternal plant-level mating system estimates (Maternal homozygosity by loci, *HL*; biparental inbreeding, *t*_*m*_*-t*_*s*_; outcrossing rate, *t*_*m*_ and correlated paternity *r*_*p*_). Pearson’s correlation coefficients shown.
(PDF)

S2 Table Individual and overall (SE) genetic parameters (maternal homozygosity by loci and mating system parameters) computed for the selected maternal plants. (PDF)

S3 Table Progeny performance of *Myrtus communis* and *Pistacia lentiscus* under greenhouse and field conditions. Data are given as means ± SE.
(PDF)
