# Supplementary material for: High Correlated Paternity Leads to Negative Effects on Progeny Performance in Two Mediterranean Shrub Species
Source: PLoS One. 2016 Nov 11;11(11):e0166023. doi: 10.1371/journal.pone.0166023 (PMC5106039; doi:10.1371/journal.pone.0166023)
Supplement: S1 Table — Pearson’s correlation coefficients shown. (PDF) [file pone.0166023.s001.pdf]

S1 Table. Correlations between correlated paternity estimates (r_p) and other mating system estimates (Maternal homozygosity, *HL*; Biparental inbreeding, t_m-t_s and outcrossing rate, t_m). Pearson's correlation coefficient shown.

	HL	t_m-t_s	t_m
Myrtus communis	-0.02^{ns}	0.38^{ns}	-0.27^{ns}
Pistacia lentiscus	0.13^{ns}	0.19^{ns}	
