# Supplementary material for: High Correlated Paternity Leads to Negative Effects on Progeny Performance in Two Mediterranean Shrub Species
Source: PLoS One. 2016 Nov 11;11(11):e0166023. doi: 10.1371/journal.pone.0166023 (PMC5106039; doi:10.1371/journal.pone.0166023)
Supplement: S2 Table — (PDF) [file pone.0166023.s002.pdf]

S2 Table. Individual (SE) genetic parameters (maternal homozygosity by loci and mating system parameters) computed for the selected maternal plants.

	Maternal homozygosity by loci (*HL*)	Mating system parameters		
		Outcrossing rate (t_m)	Biparental inbreeding (t_m-t_s)	Correlated paternity (r_p)
Myrtus communis				
M01	0.18	0.46 (0.03)	0.17 (0.02)	0.46 (0.09)
M02	0.24	0.68 (0.03)	0.38 (0.02)	0.33 (0.06)
M03	0.25	0.82 (0.03)	0.34 (0.02)	0.46 (0.07)
M04	0.25	0.69 (0.03)	0.32 (0.02)	0.32 (0.09)
M05	0.26	0.83 (0.03)	0.35 (0.02)	0.21 (0.06)
M06	0.26	0.82 (0.03)	0.22 (0.02)	0.26 (0.04)
M07	0.31	0.38 (0.03)	0.18 (0.02)	0.29 (0.14)
M08	0.32	0.82 (0.03)	0.15 (0.02)	0.32 (0.05)
M09	0.36	0.90 (0.03)	0.41 (0.02)	0.40 (0.04)
M10	0.38	0.25 (0.03)	0.08 (0.01)	0.53 (0.16)
M11	0.40	0.73 (0.03)	0.35 (0.02)	0.60 (0.05)
M12	0.40	0.98 (0.03)	0.29 (0.04)	0.20 (0.07)
M13	0.40	0.78 (0.03)	0.46 (0.03)	0.87 (0.03)
M14	0.45	0.63 (0.03)	0.14 (0.02)	0.37 (0.05)
M15	0.57	0.58 (0.03)	0.26 (0.02)	0.67 (0.06)
M16	0.59	0.76 (0.03)	0.27 (0.02)	0.23 (0.06)
M17	0.63	0.77 (0.03)	0.31 (0.02)	0.49 (0.05)
M18	0.69	0.82 (0.03)	0.07 (0.02)	0.10 (0.11)
Pistacia lentiscus				
P01	0.00	0.98 (0.00)	0.06 (0.00)	0.05 (0.00)
P02	0.32	0.94 (0.01)	0.03 (0.01)	0.05 (0.00)
P03	0.37	0.98 (0.00)	0.06 (0.00)	0.04 (0.00)
P04	0.05	0.94 (0.01)	0.05 (0.01)	0.05 (0.00)
P05	0.15	0.96 (0.00)	0.08 (0.01)	0.06 (0.01)
P06	0.15	0.99 (0.00)	0.06 (0.00)	0.03 (0.00)
P07	0.29	0.99 (0.00)	0.06 (0.00)	0.08 (0.01)
P08	0.26	0.99 (0.00)	0.06 (0.00)	0.04 (0.00)
P09	0.37	0.99 (0.00)	0.08 (0.00)	0.04 (0.00)
P10	0.34	0.99 (0.00)	0.10 (0.01)	0.10 (0.01)
P11	0.42	0.99 (0.00)	0.07 (0.00)	0.04 (0.00)
P12	0.22	0.99 (0.00)	0.07 (0.00)	0.05 (0.00)
P13	0.46	0.93 (0.01)	0.03 (0.01)	0.05 (0.00)
P14	0.39	0.99 (0.00)	0.11 (0.01)	0.10 (0.01)
P15	0.32	0.98 (0.00)	0.06 (0.00)	0.10 (0.02)
P16	0.49	0.99 (0.00)	0.10 (0.01)	0.05 (0.00)
P17	0.23	0.92 (0.02)	0.02 (0.01)	0.11 (0.01)
P18	0.05	0.99 (0.00)	0.08 (0.00)	0.05 (0.00)
