# Supplementary material for: High Correlated Paternity Leads to Negative Effects on Progeny Performance in Two Mediterranean Shrub Species
Source: PLoS One. 2016 Nov 11;11(11):e0166023. doi: 10.1371/journal.pone.0166023 (PMC5106039; doi:10.1371/journal.pone.0166023)
Supplement: S3 Table — Data are given as means ± SE. (PDF) [file pone.0166023.s003.pdf]

S3 Table. Progeny performance of *Myrtus communis* and *Pistacia lentiscus* under greenhouse and field conditions. Data are given as means ± SE.

Fitness measurements	*Myrtus communis*	*Pistacia lentiscus*
**Greenhouse experiment**		
Emergence (%)	53.33 ± 2.26	49.17 ± 1.73
Emergence time (days)	20.04 ± 0.40	18.90 ± 0.40
Seedling height (mm)		
After 36 days	16.70 ± 0.75	26.89 ± 0.78
After 113 days	136.56 ± 5.15	212.90 ± 5.41
After 190 days	220.00 ± 8.60	354.44 ± 9.02
After 267 days	230.39 ± 8.90	368.37 ± 9.35
After 424 days	257.50 ± 9.30	391.60 ± 9.80
Dry biomass (g)		
Total	2.39 ± 0.04	2.87 ± 0.04
Shoot	1.28 ± 0.03	1.58 ± 0.03
Root	1.11 ± 0.01	1.30 ± 0.01
**Field experiment**		
Emergence (%)	18.22 ± 1.86	49.07 ± 1.90
under *Myrtus*	24.24 ± 2.73	47.22 ± 3.73
under *Pistacia*	14.44 ± 2.87	46.11 ± 3.73
open ground	15.66 ± 2.73	53.89 ± 3.73
Emergence time (days)	39.19 ± 0.95	34.24 ± 0.60
under *Myrtus*	39.94 ± 1.70	35.52 ± 0.93
under *Pistacia*	34.50 ± 2.31	36.35 ± 0.91
open ground	41.97 ± 2.11	31.31 ± 0.86
Lifetime (days)	23.44 ± 0.11	94.50 ± 6.38
under *Myrtus*	18.92 ± 3.78	98.02 ± 12.41
under *Pistacia*	25.23 ± 5.14	38.28 ± 12.64
open ground	28.97 ± 4.70	138.00 ± 11.62
Survival (%)	-	15.15 ± 2.21
under *Myrtus*	-	15.29 ± 3.77
under *Pistacia*	-	2.44 ± 3.84
open ground	-	25.77 ± 3.53
